# Steps to Health in Cognitive Aging: Effects of Physical Activity on Spatial Attention and Executive Control in the Elderly

**DOI:** 10.3389/fnhum.2017.00107

**Published:** 2017-03-06

**Authors:** Giancarlo Condello, Roberta Forte, Simone Falbo, John B. Shea, Angela Di Baldassarre, Laura Capranica, Caterina Pesce

**Affiliations:** ^1^Sport Performance Laboratory, Department of Movement, Human and Health Sciences, Italian University of Sport and Movement “Foro Italico”Rome, Italy; ^2^Exercise and Cognition Laboratory, Department of Movement, Human and Health Sciences, Italian University of Sport and Movement “Foro Italico”Rome, Italy; ^3^Ergonomics Laboratory, School of Public Health, Indiana University BloomingtonBloomington, IN, USA; ^4^Department of Medicine and Aging Sciences, School of Medicine and Health Sciences, “G. d’Annunzio” University of Chieti-PescaraChieti, Italy

**Keywords:** attentional networks, reaction time, active lifestyle, late middle-aged, old, diabetes

## Abstract

The purpose of this study was to investigate whether physical activity (PA) habits may positively impact performance of the orienting and executive control networks in community-dwelling aging individuals and diabetics, who are at risk of cognitive dysfunction. To this aim, we tested cross-sectionally whether age, ranging from late middle-age to old adulthood, and PA level independently or interactively predict different facets of the attentional performance. Hundred and thirty female and male individuals and 22 adults with type 2 diabetes aged 55–84 years were recruited and their daily PA (steps) was objectively measured by means of armband monitors. Participants performed a multifunctional attentional go/no-go reaction time (RT) task in which spatial attention was cued by means of informative direct cues of different sizes followed by compound stimuli with local and global target features. The performance efficiency of the orienting networks was estimated by computing RT differences between validly and invalidly cued trials, that of the executive control networks by computing local switch costs that are RT differences between switch and non-switch trials in mixed blocks of global and local target trials. In regression analyses performed on the data of non-diabetic elderlies, overall RTs and orienting effects resulted jointly predicted by age and steps. Age predicted overall RTs in low-active individuals, but orienting effects and response errors in high-active individuals. Switch costs were predicted by age only, with larger costs at older age. In the analysis conducted with the 22 diabetics and 22 matched non-diabetic elderlies, diabetic status and daily steps predicted longer and shorter RTs, respectively. Results suggest that high PA levels exert beneficial, but differentiated effects on processing speed and attentional networks performance in aging individuals that partially counteract the detrimental effects of advancing age and diabetic status. In conclusion, adequate levels of overall PA may positively impinge on brain efficiency and attentional control and should be therefore promoted by actions that support lifelong PA participation and impact the built environment to render it more conducive to PA.

## Introduction

The rectangularization of the life expectancy curve and the increasing proportion of ‘graying’ population (Spirduso et al., 2005) urges societies toward a more comprehensive understanding of how to ensure health and quality of life of aging people. Awareness is increasing that physical activity (PA) is one of the major lifestyle-related health determinants with benefits that go beyond physical health also in advanced age (Netz et al., 2005; World Health Organization, 2007; Ballesteros et al., 2015). Over the past decades, there has been a rise and fall of interest for the different facets of PA-elicited health outcomes due to reasons ranging from epidemiological trends to methodological advancements. The worldwide overweight and insulin resistance epidemic has led to advocate for PA in aging with the aim to ensure health-appropriate levels of PA and caloric expenditure (Ryan, 2010). On the other hand, methodological advancements in cognitive and especially neuroscientific research have allowed to accumulate evidence on the beneficial impact of PA on several aspects of brain health and cognitive efficiency in the aging population with or without chronic diseases (Bherer et al., 2013; Prakash et al., 2015; Young et al., 2015; Gajewski and Falkenstein, 2016).

Age-related chronic diseases are frequently associated with cognitive impairment. Population aging appears to be the most important demographic change to the prevalence of diabetes, one of the four main types of non-communicable diseases across the world (World Health Organization, 2015) projected to double from 2000 to 2030 (Wild et al., 2004) and demonstrated to be a risk factor for cognitive decline and dysfunction as early as middle-age (Kodl and Seaquist, 2008; Nooyens et al., 2010; Luchsinger, 2012; Umegaki, 2014). Thus in recent years, the focus of PA interventions has been extended from sole physical to brain health effects. Designed, structured PA interventions (Espeland et al., 2016), as well as physically active habits as simply walking (Yaffe et al., 2001; Abbott et al., 2004) seem beneficial to counteract cognitive aging of older adults with and without diabetes, even though health-related covariates may limit effect size (Devore et al., 2009).

Echoing the title of a European framework to promote PA for health (“Steps to health,” World Health Organization, 2007), we extend the notion of the health-enhancing effects of PA to an aspect of cognitive health in aging people – the efficiency of attentional control – that has still received scarce consideration in research on the influence of PA on cognition. Particularly, the present study investigates the relation of PA habits of aging individuals with and without diabetes, as objectively assessed in terms of daily steps, to the efficiency of the attentional systems responsible for the orienting of attention and the executive control, which seem to undergo different trajectories of age-related deterioration from middle-age to older adulthood (Zhou et al., 2011).

Recent advancements in neurosciences suggest that these attentional systems rely on two interactive, but anatomically distinct networks each (Dosenbach et al., 2008; Petersen and Posner, 2012; Vossel et al., 2014). The orienting of attention is handled by both a more dorsal and a ventral network that act cooperatively to enable individuals to flexibly control attention in relation to top-down goals and bottom-up sensory stimulation (Corbetta and Shulman, 2002; Vossel et al., 2014). The dorsal network, including parietal regions, as the intraparietal sulcus, but also a small set of frontal locations, particularly in the frontal eye fields, allows for strategic control over attention according to the information delivered by environmental cues. The ventral network, including the ventral frontal cortex and the temporoparietal junction, comes into play when the focus of attention is erroneously engaged by misleading cues and must be therefore disengaged and shifted in a task-relevant direction (Petersen and Posner, 2012).

Executive control is guaranteed by the interplay of two distinct networks too: the fronto-parietal and the cingulo-opercular components. The first seems responsible for the adaptability, the second for the stability of top-down task control (Dosenbach et al., 2008). Particularly, the fronto-parietal network, including lateral frontal and parietal regions and particularly the dorsolateral prefrontal cortex, is supposed to initiate executive control and handle its ongoing adjustment for conflict resolution on a trial-by-trial basis. The cingulo-opercular network, including the anterior cingulate cortex, the anterior insula and frontal regions as the frontal operculum, appears to ensure a stable ‘set maintenance’ over trials by monitoring the preparatory allocation of attention especially in presence of competing attentional sets (Luks et al., 2002; Petersen and Posner, 2012).

After the seminal meta-analysis by Colcombe and Kramer (2003), showing that executive functions of older adults are more improved by PA than lower-level functions, researchers have confirmed such larger or selective effects and devoted noticeable efforts to further differentiate PA effects on specific aspects of executive vs. non-executive function (Gajewski and Falkenstein, 2016). Executive function is responsible for crucial aspects of cognition as planning of goal-oriented actions, monitoring of cognitive operations and behavioral adaptability (Diamond, 2013). Thus, this special focus on PA effects on executive function is well justified, but has led, with specific regard to the PA-attention relationship, to a disproportional interest for the attentional networks responsible for executive control and to a relative neglect of the other attentional networks the executive control network is strictly intertwined with. The study of PA effects on attentional orienting is mainly limited to the effects of acute bouts of exercise in young active adults (Pesce et al., 2007b; Huertas et al., 2011; Sanabria et al., 2011; Luque-Casado et al., 2013; Chang et al., 2015; Llorens et al., 2015). The only two attentional orienting studies performed with older adults have tested the moderation of acute exercise effects by chronic PA participation (Pesce et al., 2007a, 2011).

The lack of aging studies that examine the separate and joint effects of chronic PA on the orienting and executive control networks is surprising, because such networks contribute in an intertwined manner to the ability to allocate attention in response to expectations and environmental stimuli (Petersen and Posner, 2012) that is relevant to functioning and safety of older adults in everyday life (Bédard et al., 2006). Several situations, as in house hold or road traffic, require the ability to decide where and what to pay attention in advance, as a traffic light for pedestrians, coupling go/no-go actions, but also to re-orient attention rapidly to changes in the environment, as a car unexpectedly approaching, or selecting from the set of possible locomotor actions the most adequate to key features of the situation, avoiding distraction from other irrelevant sources.

Thus, the primary aim of the present study was to investigate whether active PA habits have a similar or differentiated impact – if any – on the performance of the orienting and executive control networks in aging. Of the large body of cognitive research that has investigated the interplay between age and PA (Young et al., 2015; Gajewski and Falkenstein, 2016), most studies have used age and PA levels as categorical variables. When, rarely, direct/indirect measures of PA were used as continuous predictors (e.g., Bixby et al., 2007), age was accounted for as a covariate, thus neglecting the potential interaction between PA and age. Also as regards age, mostly younger and older age classes were compared, whereas the interesting transition phase of late middle-age, characterized by a unique interplay between covert neural and overt behavioral changes (Berchicci et al., 2012) remains relatively underinvestigated. Since specific aspects of the attentional orienting (Pesce et al., 2007a) and executive control networks (Hillman et al., 2006; Themanson et al., 2006) seem benefited by PA at old age, we hypothesized that aging and PA level may have interactive effects on the different facets of the attentional performance across a wide range of ages from late middle-age to old adulthood.

The second aim, related to the first one, was to investigate whether aging diabetics, who are at risk of poor cognition (Luchsinger, 2012), may profit from the expected attentional benefits of being physically active. Although PA and exercise training is considered a major therapeutic modality for type 2 diabetes, persons affected by this pathology usually exhibit lower levels of PA and related lower levels of cardiovascular fitness (Albright et al., 2000). This may prevent diabetics from successful cognitive aging, which is linked to physically active habits (Young et al., 2015) through different mechanisms and above all the enhancement and maintenance of cardiovascular fitness (Stillman et al., 2016). Recent evidence suggests that the mechanisms through which PA affects cognitive function may differ for aging persons by diabetes status, since beneficial cognitive outcomes of PA were found in diabetic elderlies, but not in co-aged individuals without diabetes (Espeland et al., 2016). Thus, we hypothesized to find more pronounced benefits in diabetics than in non-diabetics. Espeland et al.’s (2016) study addressed global cognition and memory. Nevertheless, of particular concern is evidence showing that among the broad range of cognitive functions impaired by type 2 diabetes, there is executive function (Qiu et al., 2006; Okereke et al., 2008). Given the critical role that executive control plays in functional abilities relevant for everyday life of aging people (Rucker et al., 2012; Forte et al., 2013, 2015), we deemed relevant to examine if a physically active lifestyle counteracts the deterioration of the ability to exert executive control over attention in this special population.

## Materials and Methods

This study was carried out in accordance with the recommendations of “Umberto I” hospital of the First Rome University with written informed consent from all subjects. All subjects gave written informed consent in accordance with the Declaration of Helsinki. The protocol was approved by the Ethics Committee of the “Umberto I” hospital of the First Rome University.

### Participants

Hundred and thirty participants (68 females and 62 males) were recruited according to the following eligibility criteria: (i) age between 55 and 84 years; (ii) not self-reported diagnosis of psychiatric or somatic illnesses, (iii) normal or corrected-to-normal vision. Also, they were stratified sampled for age class (Spirduso et al., 2005): late middle-aged (55–64 years = 48), young-old (65–74 years = 44), and old adults (75–84 years = 38). Within each age class, they were further stratified sampled for their declared PA level to ensure a balanced presence of sedentary and physically active individuals and master athletes (runners and swimmers) engaged in regular structured physical PA/training for ≥3 (*n* = 42), ≥2 (*n* = 46), or < 2 (*n* = 42) sessions/week, respectively.

To address the secondary aim of this study, also a sample of 22 late middle-aged, young-old, and old adults with type 2 diabetes (55–64 = 5; 65–74 = 12; 75–84 = 5) was recruited. Accordingly to literature (Hu et al., 1999), a case of diabetes was considered confirmed if at least one of the following criteria was reported: (1) one or more classic symptoms (excessive thirst, polyuria, weight loss, hunger) and fasting plasma glucose levels of at least 140 mg/dL (7.8 mmol/L), or random plasma glucose levels of at least 200 mg/dL (11.1 mmol/L); (2) at least two elevated plasma glucose concentrations on different occasions [fasting levels of at least 140 mg/dL (7.8 mmol/L), random plasma glucose levels of at least 200 mg/dL (11.1 mmol/L), and/or concentrations of at least 200 mg/dL after 2 h or more shown by oral glucose tolerance testing] in the absence of symptoms; (3) treatment with hypoglycemic medication (insulin or oral hypoglycemic agent).

### Health, Physical Activity, and Anthropometric Assessment

Participants answered the AAHPERD (American Alliance for Health, Physical Education, Recreation and Dance) exercise/medical history questionnaire (Osness et al., 1996) ascertaining their activity level, educational background, dietary habits, tobacco smoking and alcohol consumption, medication use and history of PA.

Daily PA was measured under free-living conditions using the SenseWear Pro3 armband (BodyMedia, Pittsburgh, PA, USA). The use of SenseWear Pro armband has been already validated in older adults (Mackey et al., 2011). The armband is a monitor that integrates the information gathered by the two axis accelerometers and sensors (i.e., skin and near body temperature, heat flux, and galvanic skin response) with sex, age, height, weight, smoking status, and handedness of the user. It provides proprietary algorithms to give quantitative information (e.g., number of daily steps, locomotor activity intensity, and energy expenditure; Di Blasio et al., 2016) about an individual’s habitual PA involving any form of locomotion as activities at workplace, sports, conditioning, house holding. The descriptive characteristics of the participants were entered into the software program (SenseWear Professional 8; BodyMedia) before the monitoring was initialized. The participants wore the armband on the right arm over the triceps muscle at the midpoint between the acromion and olecranon processes. According to reliability criteria reported in the literature, participants wore the armband for seven entire and consecutive days, 24 h a day except during water-based activities (Scheers et al., 2012), with a wear time of at least 540 min/day on weekdays and 480 min/day on weekend days (Di Blasio et al., 2016). From the default information given by the software, the mean value of steps of 7 days was used for the statistical analysis.

Standing height to the nearest 0.1 cm and body mass to the nearest 0.1 kg, were measured using a portable stadiometer (Seca 220, GmbH & Co., Hamburg, Germany) and a balance scale (Seca 761, GmbH & Co., Hamburg, Germany), respectively. Body mass index (BMI, kg^∗^m^-2^) was computed. Background information on the participants, as main health, lifestyle and anthropometric characteristics are reported in **Table 1** separately for non-diabetic and diabetic individuals and for the three age classes (late middle-aged, young-old, old).

**Table 1:** Background characteristics of the participants: gender, anthropometric data, steps, education, number of medications and diseases, retirement, smoking, and alcohol habits.

	Non-diabetics (*n* = 130)			Diabetics (*n* = 22)		
	Late middle-aged (55–64 years)	Young-old (65–74 years)	Old (75–84 years)	Late middle-aged (55–64 years)	Young-old (65–74 years)	Old (75–84 years)
**Gender**						
Female (*n*)	25	21	16	1	4	1
Male (*n*)	23	23	22	4	8	4
Height (m)	1.68 ± 0.09	1.64 ± 0.09	1.62 ± 0.09	1.66 ± 0.03	1.64 ± 0.08	1.69 ± 0.03
Body mass (kg)	75.2 ± 14.4	72.2 ± 12.4	68.3 ± 9.6	88.2 ± 13.0	78.6 ± 13.6	78.9 ± 8.5
BMI (kg/m^2^)	26.4 ± 3.8	26.9 ± 3.4	26.1 ± 3.4	32.0 ± 6.0	29.1 ± 4.0	27.5 ± 2.9
**Step** (mean)	11488 ± 3473	11285 ± 3847	9146 ± 3401	9354 ± 3878	9402 ± 3823	6801 ± 1286
**Educational level**						
<High school (*n*)	6	12	14	4	7	1
High school (*n*)	25	24	15	1	4	1
College (*n*)	17	8	9	0	1	3
**Drugs** (*n*)	2.0 ± 1.9	3.0 ± 2.6	3.8 ± 3.4	3.8 ± 3.0	4.4 ± 2.4	5.2 ± 1.9
**Diseases** (*n*)	1.0 ± 1.4	2.5 ± 2.1	3.4 ± 2.5	6.6 ± 3.9	4.6 ± 2.5	5.6 ± 2.6
**Retirement**						
Yes (*n*)	21	41	34	2	10	5
No (*n*)	27	3	4	3	2	0
**Smoking**						
No (*n*)	20	25	20	1	5	3
In the past (*n*)	19	16	13	2	6	2
Yes (*n*)	9	3	5	2	1	0
**Alcohol**						
No (*n*)	22	12	14	2	3	3
Occasionally (*n*)	26	32	24	3	9	2

### Attentional Assessment

The attentional test, developed by Pesce et al. (2003) by means of the Experimental Run Time System (ERTS, BeriSoft Cooperation), has been applied in aging research to investigate the effects of acute bouts of exercise and those of chronic PA participation on performance of the attentional networks responsible for orienting (Pesce et al., 2007a, 2011) and executive control (Pesce and Audiffren, 2011). The testing took place either in the morning or in the afternoon, according to participants’ availability, avoiding the time before 9 am, between 1 and 3 pm, and after 7 pm to minimize undesired reaction time (RT) variability due to circadian vigilance fluctuation.

#### Apparatus and Stimuli

Participants were seated in a dimly lit room at a distance of 60 cm from a PC-driven video screen. Four visual displays were used: the instruction, presented on the screen only one time at the beginning of the experimental session, and three types of stimuli, sequentially presented on the screen at each trial. They were a central fixation point, a spatial cue of variable size, and a compound stimulus. The fixation point was a tilted “T” of 0.4° × 0.4° and the spatial cue was an empty box of 1° × 1° or 5° × 5°. The compound stimulus was a large letter (4.6° × 4.6°) made of 13–17 small letters (0.6° × 0.6°) spaced 0.4° in a 5 × 5 matrix. The large letter and its small elements represented the global and local level of the compound stimulus, respectively. The large letter could be an A, E, F, or H; the small elements were the remaining letters. The fixation point, the large box and the following compound stimulus were centered on the screen; the small box could randomly appear at one of the locations of the elements composing the compound stimulus.

#### The Attentional Task

**FIGURE 1:**
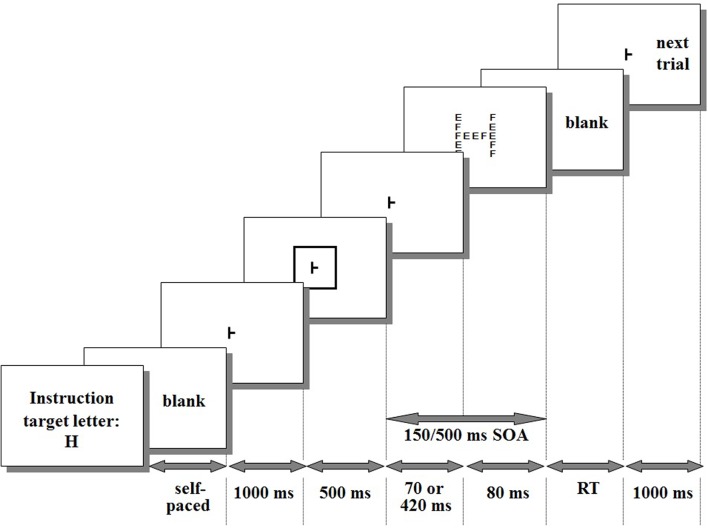
**Timing of the event sequence within a trial**. As an example, the cue is large and the target letter (“H”) matches in size with the cue.

**FIGURE 2:**
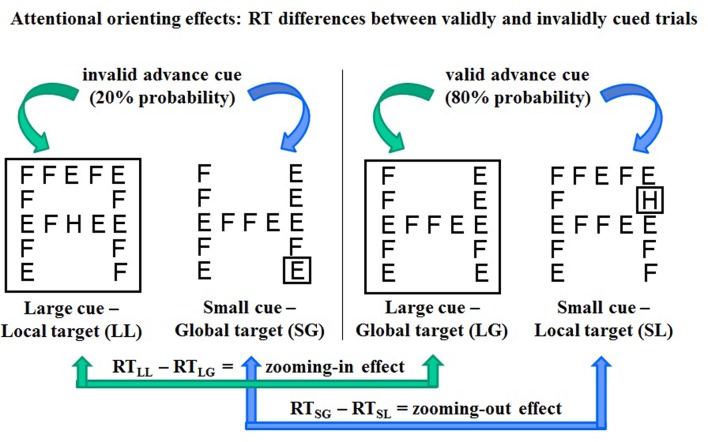
**Schematic representation of four types of trials (with “H” as target letter for example)**. Left: invalidly cued trials with cue-target mismatching, right: validly cued trials with cue-target matching. Bottom: computation of RT differences between invalidly and validly cued trials as estimates of attentional zooming in (right) and zooming out (left) effects.

Each trial consisted of the sequence of events represented in **Figure 1**. In five sixths of the trials (go trials), the compound stimulus contained a target letter (e.g., “H,” **Figures 1**, **2**) either at the global or at the local level. Participants had to react as soon as possible to the target letter by pressing a RT-key with the right index finger while gazing at the fixation point. In the remaining trials (no-go trials), the compound stimulus did not contain the target letter and participants had to refrain from responding. Responses to no-go trials or responses with RTs shorter than 200 ms or longer than 2,500 ms were considered errors (anticipations and delayed responses, respectively) and were discarded. The response caused the offset of the compound stimulus for the next trial to begin after an inter-trial interval of 1,000 ms.

In 80% of the go trials, the size of the cue and that of the upcoming target were matched: a large cue was followed by a global target and a small cue by a local target at the same location (validly cued trials, **Figure 2** left). In the remaining 20% of trials, cue and target size were mismatched (invalidly cued trials, **Figure 2** right). Before the experiment, participants were instructed to focus their attention on the area of the visual field delimited by the spatial cue, without shifting their gaze, in order to react as soon as possible to a predefined target letter that would probably match cue size. Further instructions were aimed at forcing participants, in the case of cue-target mismatching, to directly switch from the global to the local level (attentional zooming in) or from the local to the global level (zooming out), avoiding visual search strategies. It was explained that when a large cue was not followed by a global target letter, the target was the local letter at the center of the screen; when a small cue was not followed by a local target letter at the cued location, the target was the global letter (**Figure 2**, right).

There were two blocks of 76 trials, one with short (150 ms) time interval between the onset of the cue and the onset of the target stimulus that follows (cue-target Stimulus-Onset-Asynchrony, SOA) and one with long (500 ms) SOA, lasting 3–4 min depending on SOA and reaction speed of the participant. Each block included four warm-up trials, 60 go trials and 12 no-go trials. Short and long SOAs were blocked and not randomized within blocks to avoid a bias in target expectancy. If SOAs were randomized within blocks, the probability (and therefore expectancy) of the target would increase after the short SOA was passed without target occurrence.

Testing was preceded by one block of practice trials to ensure that set acquisition reached a learning asymptote in both younger and older individuals. The minimum amount of practice (40 trials) could be automatically prolonged until a criterion frequency of 80% correct responses was reached. The order of the two blocks of trials with short and long SOA within each task, as well as the use of two of four possible target letters were counterbalanced across participants. Also, to reduce potential threats to internal validity deriving from the use of four different letters, all possible combinations with the remaining non-target letters at the global and local level of the compound stimuli were balanced and randomized within blocks. Cue sizes and target levels were balanced and randomized within blocks. Particularly, the 50% frequency of global or local target occurrence allowed to balance the priming effects between consecutive global- or local-target trials (Robertson, 1996) that were estimated as a measure of switch costs (see “Switch Costs”).

## Preliminary Computations and Analyses

Trials with response errors (responses with RTs shorter than 200 ms or longer than 2,500 ms) were discarded and median RTs were computed for correct trials separately for each type of trial. Median instead of mean RTs were used because of the disproportional contribution of outliers on mean RTs and the appropriateness of median values for positively skewed distributions, as RTs usually are, as long as RT differences, not absolute RTs, are relevant (Pesce et al., 2003). Thus, computations of RT differences of interest were performed on median RT data of correct trials to isolate the performance of the (i) orienting and (ii) executive control networks from the performance of the processing systems that, handling incoming stimuli and producing outputs, contribute to general information processing speed. Specifically, we computed (i) RT differences that reflect the efficiency of the exogenous (automatic) and endogenous (intentional) control of attentional orienting (Lauwereyns, 1998) and (ii) switch costs that reflect how an individual is able to cope with the cognitive flexibility requirements of the attentional task (Rogers and Monsell, 1995).

### Attentional Orienting Effects

As common in spatial cueing paradigms (Chica et al., 2014), attentional orienting effects were generated by manipulating the validity of the spatial cue: cue and target size where most probably matching and only rarely mismatching (80 and 20% probability, respectively). Consequently, participants were expected to react faster on validly cued trials with targets matching in size the antecedent cue (**Figure 2**, left) and slower on invalidly cued trials with cue-target mismatching (**Figure 2**, right). To estimate the time needed to refocus attention when a misleading cue leads to focus attention at the wrong spatial scale, RT differences between invalidly and validly cued trials were computed as follows (**Figure 2**):

1. RT_(smallcue_ _-globaltarget)__-_ RT_(smallcue_ _-localtarget)_ = zooming out effect
2. RT_(largecue__-__localtarget)__-_ RT_(largecue__-__globaltarget)_ = zooming in effect

The attentional task was originally designed to tap the exogenous and endogenous control of attentional orienting jointly within the same task (Pesce et al., 2003). The abrupt onset of the direct cue was expected to elicit an automatic, short-lasting orienting of attention toward the cued area affecting performance at short SOA (Stoffer, 1993; Lamb et al., 2000). The informative value of the direct cues as to where the upcoming target should occur was expected to generate a lower-rising spatial expectancy affecting performance especially at longer SOA. Traditional views attributed the exogenous, stimulus-driven and the endogenous, intentional control of attention allocation to the ventral and dorsal networks, respectively. In recent years, this dichotomy has been replaced by a more interactive view attributing to the dorsal and ventral networks a joint role in both exogenous and endogenous control of attentional orienting to locations and features (Macaluso and Doricchi, 2013; Vossel et al., 2014). Thus, to have an overall estimate of the joint activity of the two networks responsible for attentional orienting, the above RT differences were computed merging short- and long-SOA trials. Means and standard deviations of median RTs calculated for the four types of trials used for the calculation of attentional orienting effects and the RT differences that reflect zooming in/out effects are presented in **Table 2**.

**Table 2:** Means ± SD of median Reaction Times (ms) of community-dwelling non-diabetic elderlies (*n* = 130) and co-aged diabetics (*n* = 22, within brackets) calculated for the four types of validly/invalidly cued trials and reaction time (RT) differences computed to estimate spatial cueing (zooming) effects.

Type of trial	RT ± SD	RT difference
Invalidly cued SG trials	800 ± 209 (754 ± 153)	Zooming out effect: 10 ± 288 (-111 ± 227)
Validly cued SL trials	790 ± 189 (865 ± 181)
Invalidly cued LL trials	789 ± 277 (779 ± 127)	Zooming in effect: 119 ± 298 (43 ± 174)
Validly cued LG trials	669 ± 122 (736 ± 166)
All go trials	762 ± 134 (784 ± 109)

*SG, small cue-global target; LL, large cue-local target; SL, small cue-local target; LG, large cue-global target. Data are collapsed across short and long Stimulus-Onset Asynchronies (SOAs)*.

### Switch Costs

The structure of the present attentional task allowed assessing executive function by computing a classical index of cognitive flexibility and executive control of cognitive processes, labeled specific (or local) switch cost (Rogers and Monsell, 1995; Kiesel et al., 2010). Since the attentional task was composed of equally frequent trials with global or local target stimulus dimensions, presented in a random order within heterogeneous blocks, participants had to switch between global and local attending. In general terms, in tasks involving the switching between two tasks A and B within heterogeneous trial blocks, trial n + 1 may be a repetition of task A or B (A–A or B–B) or an alternation of tasks A and B (A–B or B–A). Specific switch costs are computed as the difference between the RT for repetition trials and the RT for switch trials. Especially when task switching is explicitly cued, switch costs are proven to index the duration of a true executive control process of task set reconfiguration that must suppress the proactive interference from the previous, no longer appropriate stimulus-response mapping and activate a new relevant task set (Jost et al., 2008).

**FIGURE 3:**
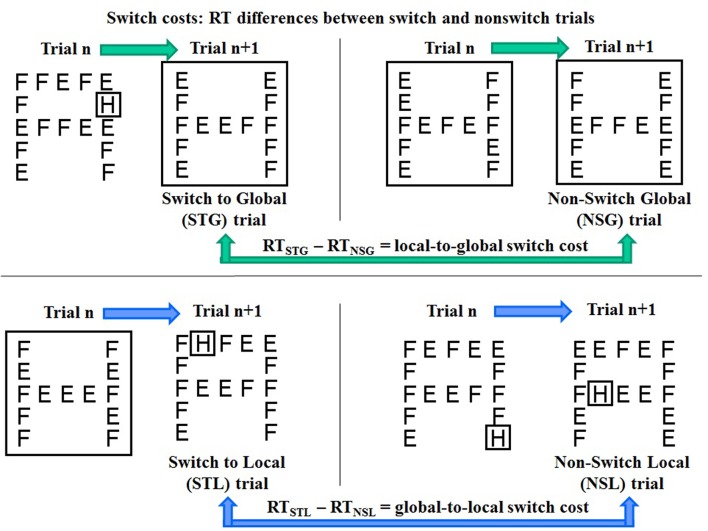
**Schematic representation of the four types of switch and non-switch trials with global and local targets (“H” in the example) and computation of RT differences between switch and non-switch trials as estimates of local to global (top) and global to local (bottom) switch costs**.

In the present experiment, switches between global and local target features of complex visual stimuli were explicitly cued by the preceding spatial cue. To isolate the switch costs from differential attention orienting effects of validly vs. invalidly cued targets, only trials with cue-target matching (80% of trials, comprising equally frequent large cue-global target and small cue-local target trials) were used for switch costs computation. Each trial was coded as “switch trial” or “non-switch trial” according to whether it was preceded by a trial with a target at the different or the same object level, respectively. Thus, four types of trials were identified (**Figure 3**):

(1) switch to global (STG, i.e., a global target trial preceded by a local target trial);
(2) non-switch global (NSG, i.e., a global target trial preceded by a global target trial);
(3) switch to local (STL, i.e., a local target trial preceded by a global target trial);
(4) non-switch local (NSL, i.e., a local target trial preceded by a local target trial).

Median RTs were computed separately for each type of trial. Means and standard deviations of median RTs calculated for the four types of trials are presented in **Table 3**. Switch costs were calculated as RT differences between switch trials and non-switch trials, representing an estimate of the time required to switch from attending to the global level of a visual object on trial n to attending to the local level on trial n + 1, or vice versa:

**Table 3:** Means ± SD of median Reaction Times (ms) of community-dwelling non-diabetic elderlies (*n* = 130) and co-aged diabetics (*n* = 22, within brackets) calculated for the four types of switch and non-switch trials used to compute RT differences as estimates of switch costs.

Type of trial	RT ± SD	RT difference
Switch to global (STG)	697 ± 187 (811 ± 210)	Switch to global: 87 ± 158 (155 ± 172)
Non-switch global (NSG)	610 ± 142 (656 ± 185)
Switch to local (STL)	754 ± 233 (837 ± 231)	Switch to local: -6 ± 156 (5 ± 165)
Non-switch local (NSL)	760 ± 223 (832 ± 235)

*Data are collapsed across SOAs*.

1. local-to-global switch cost = RT_STG_ – RT_NSG_ (**Figure 3**, top).
2. global-to-local switch cost = RT_STL_ – RT_NSL_ (**Figure 3**, bottom).

### Error Rates

Three types of error rates were calculated: real response errors (responses to no-go trials), anticipated responses (RTs shorter than 200 ms), and delayed responses (RTs longer than 2,500 ms). They were computed both as overall error rates and separately for the different types of experimental conditions used to obtain attentional orienting effects and switch costs (**Table 4**). Since anticipated responses were overall very low (2%), they were not analyzed further.

**Table 4:** Average percentage errors of community-dwelling non-diabetic elderlies (*n* = 130) and co-aged diabetics (*n* = 22, within brackets) calculated for the four types of validly/invalidly cued go trials and the two types of no-go trials.

Type of trial	Anticipated responses (%)	Delayed responses (%)	Response errors (%)
*Small-cue trials:*	21% (20%)
Invalidly cued SG trial	1% (0%)	33% (25%)
Validly cued SL trial	3% (0%)	25% (26%)
*Large-cue trials:*	20% (17%)
Invalidly cued LL trial	1% (0%)	25% (20%)
Validly cued LG trial	2% (0%)	13% (14%)
*All go trials*	2% (0%)	21% (20%)
*All no-go trials*	21% (18%)

*SG, small cue-global target; LL, large cue-local target; SL, small cue-local target; LG, large cue-global target*.

## Results

### Effects of Age and Physical Activity in Aging Individuals

The first question regarded whether the efficiency of the attentional networks responsible for attentional orienting and executive control are differentially affected by transitions from late middle-aged to young-old and old adulthood and whether physically active habits may counteract the hypothesized age-related deterioration. To address this question, RT differences of community-dwelling aging individuals, computed to estimate attentional orienting effects and switch costs, were regressed on age with daily steps as moderator and gender and BMI as covariates. The rationale for including BMI as a covariate was that we aimed at disentangling the role played by habitual PA from weight status, whose independent or joint influence on cognition across the lifespan and in aging is still an issue of debate (Memel et al., 2016; Chang et al., 2017), but goes beyond the aim of the present study.

This moderated regression model entailed the following steps: (1) computing the interaction variable by multiplying age and daily steps (after centering them); (2) performing a hierarchical multiple regression analysis for the prediction of RT by age, daily steps, and their interaction term. Gender and BMI were statistically controlled for by entering them in a first block, while the individual predictors (age and daily steps) were entered in a second block and their interaction term in a third block. (3) In case, the interaction term significantly predicted RT, *post hoc* analysis through simple slope test was performed (Aiken and West, 1991). The statistical significance was set at *p* < 0.05.

Also overall reaction speed (absolute RTs), as well as accuracy (response errors and delayed responses) data were submitted to the same regression analysis models. This allowed ensuring that larger RT differences of interest would not be merely due to longer RTs in absolute terms, or that smaller RT differences would merely reflect a shift in speed-accuracy trade-off setpoint. For instance, if older adults would show, as expected, longer RTs, this might lead to proportionally larger zooming effects and switch costs, which are RT differences. If such longer RTs and correspondently larger RT differences would be paralleled by lower rates of responses to no-go trials, it might be just due to the fact that older individuals traded speed for accuracy.

Since no reference data for *a priori* power analysis for multiple regression were available from aging studies with the employed attentional variables, *post hoc* achieved power (1-β) was computed with the G^∗^Power program (Faul et al., 2009).

#### Reaction Speed

**FIGURE 4:**
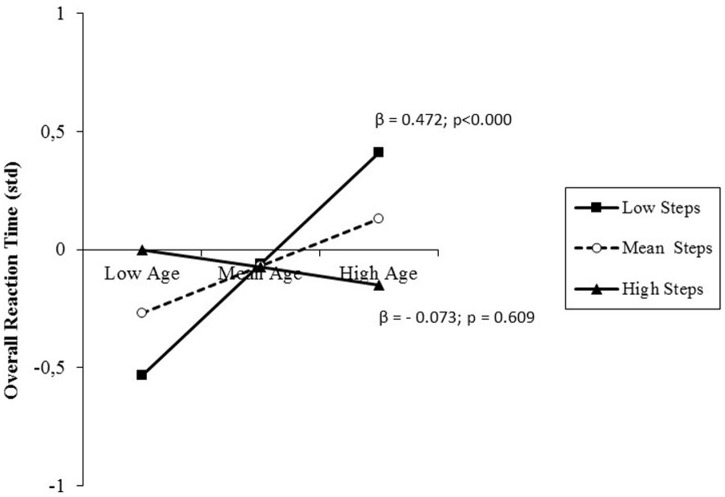
**prediction of overall reaction time (RT) accrued by age and moderated by physical activity (PA) level (daily steps)**. Solid lines: change in the slope of the predictor for high vs. low PA levels (1 SD change); β values and their significance are also reported.

The results of the analysis performed on overall RT are presented in **Table 5**, females (744 ± 126 vs. 782 ± 141 ms) and a direct relationship between age and RT, indicating that RT slows down with increasing age. However, there was a further small, but significant percentage of variance explained by the interaction between age and daily steps, suggesting that the effect of age on RT was moderated by the activity level. Simple slope testing (**Figure 4**) showed a buffering effect of the moderator on the predictor. While in low-active adults, older age predicted longer RT, this negative effect of age on RT was not present in high-active adults along the entire age range from late middle-age to old adulthood. *Post hoc* observed power (1-β) was 0.99.

**Table 5:** Hierarchical regression models testing moderated prediction of overall RT, attentional orienting (zooming) effects, and switch costs in community-dwelling elderlies (*n* = 130).

	Overall RT			Zooming effects			Switch costs		
	Beta (Std.)	*t*	*p*	Beta (Std.)	*t*	*p*	Beta (Std.)	*t*	*p*
**Factors in block 1**									
Gender	0.69	-2.24	0.027	n.s.	n.s.	n.s. (out)	n.s.	n.s.	n.s. (LtG)
				n.s.	n.s.	n.s. (in)	n.s.	n.s.	n.s. (GtL)
Body Mass Index	n.s.	n.s.	n.s.	n.s.	n.s.	n.s. (out)	n.s.	n.s.	n.s. (LtG)
				n.s.	n.s.	n.s. (in)	n.s.	n.s.	n.s. (GtL)
***R*^2^**			0.05			0.02 (out)			0.01 (LtG)
					<0.01 (in)			0.01 (GtL)
**Factors in block 2**									
Age	0.17	1.96	0.053	-0.35	-3.88	<0.001 (out)	0.23	2.41	0.018 (LtG)
				-0.26	-2.78	=0.006 (in)	n.s.	n.s.	n.s. (GtL)
Daily steps	n.s.	n.s.	n.s.	n.s.	n.s.	n.s.	n.s.	n.s.	n.s. (LtG)
							n.s.	n.s.	n.s. (GtL)
***R*^2^ change**			0.05			0.09 (out)			0.05 (LtG)
					0.06 (in)			0.02 (GtL)
**Factor in block 3**									
Age × Daily steps	-0.27	-3.14	0.002	-0.20	-2.35	0.020 (out)	n.s.	n.s.	n.s. (LtG)
				n.s.	n.s.	n.s. (in)	n.s.	n.s.	n.s. (GtL)
***R*^2^ change**			0.05			0.04 (out)			<0.01 (LtG)
					<0.01 (in)			<0.01 (LtG)
			Total *R*^2^ = 0.17			Total *R*^2^ = 0.14 (out)			Total *R*^2^ = 0.06 (LtG)
					=0.07 (in)			=0.03 (GtL)

*Total *R*^2^ explained and standardized β coefficients with *t*-values and significance level are reported. out, in = spatial attentional zooming out or zooming in effects; LtG, GtL = local-to-global or global-to-local switch costs*.

#### Attentional Orienting Effects

**FIGURE 5:**
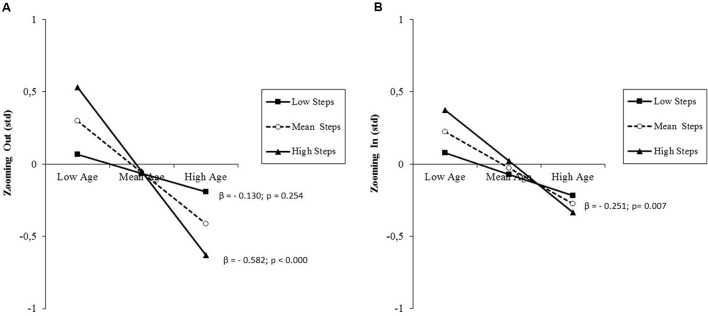
**(A)** Prediction of attentional zooming out effects accrued by age and moderated by PA level (daily steps); **(B)** prediction of attentional zooming in effects accrued by age without any significant moderation by PA level. Solid lines: change in the slope of the predictor for high vs. low PA levels (1 *SD* change); β values and their significance are reported for each single slope of the moderated prediction **(A)**, or for the main slope (dotted line) of the non-moderated prediction **(B)**.

The results of the analysis performed on attentional orienting effects are presented in **Table 5**, middle, separately for the two directions of the attentional zooming. This distinction was deemed necessary because the size of the two effects differed greatly, with zooming out effects being averagely almost absent with a huge interindividual variability (**Table 2**). Regardless of zooming direction, there was a significant prediction by age. Additionally for zooming out, there was a further small, but significant percentage of variance explained by the interaction between age and daily steps. In contrast to what observed in the case of overall RT, simple slope testing showed an inverse relationship between age and the size of the attentional zooming effect and an amplifying effect of the moderator (**Figure 5A**). While low-active adults showed an averagely almost absent zooming out effect regardless of age, high-active adults showed such effect, but with an age-related decrement from late middle-aged to old adulthood. Visual inspection of single slopes showed a similar, but non-significant pattern of results for zooming in effects (**Figure 5B**). *Post hoc* observed power (1-β) from the analysis of zooming out and zooming in effects was 0.98 and 0.88, respectively.

#### Switch Costs

**FIGURE 6:**
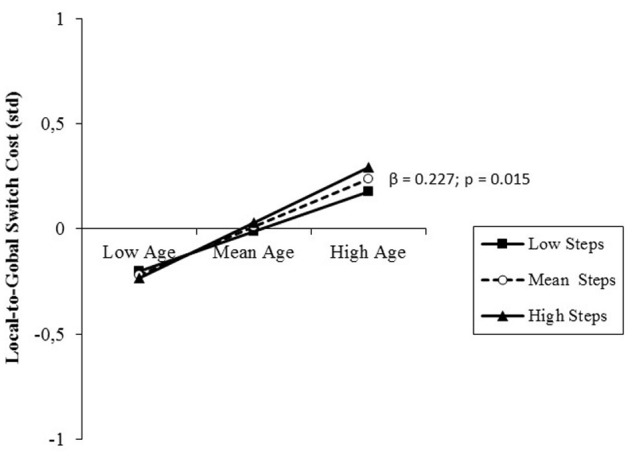
**Prediction of local-to-global switch costs accrued by age without any significant moderation by PA level (daily steps)**. Solid lines: non-significant change in the slope of the predictor for high vs. low PA levels (1 *SD* change); β value and its significance are reported for the main slope (dotted line) of the non-moderated prediction.

The results of the analysis performed on switch costs are presented in **Table 5**, right, separately for the two directions of local-to-global and global-to-local switches. Similar to what explained for the zooming effects, this distinction was deemed necessary also for switches of attention between global and local features of visual objects. Also in this case, the effect in one switch direction was averagely not detectable (i.e., small negative value, **Table 3**). Results of the regression analysis evidenced only a small, but significant prediction by age of local-to-global switch costs, with increasing switch costs at older age. This direct relationship was not moderated by PA level (**Figure 6**). *Post hoc* observed power (1-β) from the analysis of local-to-global and global-to-local switch costs was 0.82 and 0.54, respectively.

#### Error Rates

**FIGURE 7:**
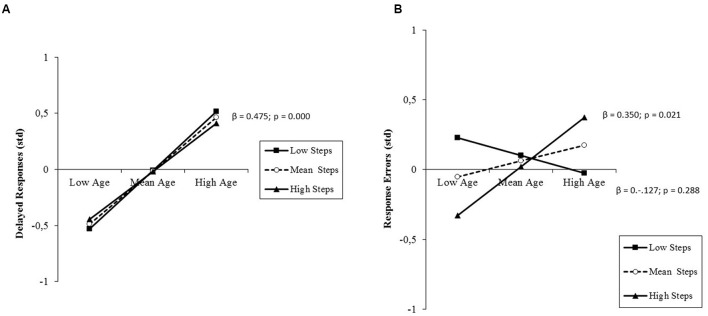
**(A)** Prediction of delayed responses to go trials accrued by age without any significant moderation by PA level (daily steps); **(B)** prediction of response errors (responses to no-go trials) accrued by age and moderated by PA level. Solid lines: change in the slope of the predictor for high vs. low PA levels (1 *SD* change); β values and their significance are reported for the main slope (dotted line) of the non-moderated prediction **(A)**, or for each single slope of the moderated prediction **(B)**.

The same model of regression analysis performed on delayed responses yielded a large percentage of variance explained by age (*R*^2^ = 0.24, std β = 0.49, *t* = 5.86, *p* < 0.001). The older the person, the larger the amount of delayed responses (**Figure 7A**). This age effect was not moderated by PA level, whereas an interactive prediction by age and PA level emerged from the analysis of response errors (*R*^2^ = 0.10, std β = 0.23, *t* = 2.62, *p* = 0.010). Simple slope testing (**Figure 7B**) showed that high-active adults, as compared to their low-active counterparts, had lower rates of responses to no-go trials at late middle-age, but higher rates at old adulthood, due to the presence of an incremental trend as a function of age in high-active participants only. *Post hoc* observed power (1-β) from the analysis of delayed responses and response errors was 1.0 and 0.97, respectively.

### Effects of Diabetic Status and Physical Activity in Aging Individuals

The second question of the present study regarded whether the diabetic status affects the cognitive functions of interest and PA level may buffer diabetes-related cognitive impairments. To address this question, further regression analyses were performed on the same dependent variables, but contrasting the data of the 22 late middle-aged, young-old, and old adults with type 2 diabetes recruited for this study with those of a subsample of 22 non-diabetic individuals selected from the main sample. Matching criteria for selection were: gender, age (± 1 year), and mean number of daily steps closest to that of the diabetic and non-diabetic participants (correlation between daily steps of age- and gender-matched pairs of diabetic participants: *r* = 0.96, *p* < 0.001). By matching diabetics and non-diabetics for daily steps, we aimed at isolating the hypothesized attentional differences due to the diabetic status from those expectedly due to lower PA levels and related lower fitness in diabetics (Albright et al., 2000), according to the cardiovascular fitness hypothesis of chronic PA effects on cognition (Stillman et al., 2016). In a moderated regression model, BMI was statistically controlled for by entering it in a first block, while the individual predictors (diabetic/non-diabetic status and daily steps) were entered in a second block and their interaction term in a third block.

#### Reaction Speed

**FIGURE 8:**
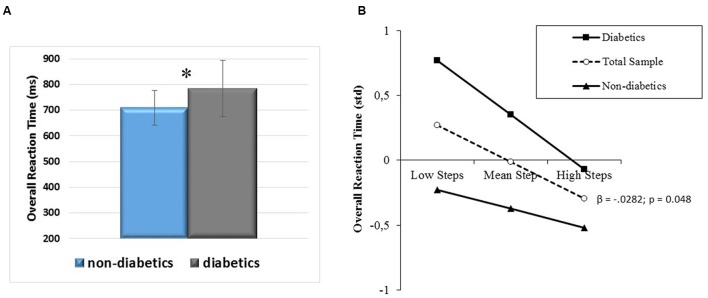
**(A)** Overall RT differences between diabetic and non-diabetic aging individuals (mean ± SD) and **(B)** prediction of overall RT accrued by PA level (daily steps) independently of diabetic/non-diabetic status. Solid lines: change in the slope of the predictor for diabetic vs. non-diabetic aging individuals; β values and their significance are reported for the main slope (dotted line) of the non-moderated prediction. ^∗^*p* < 0.05.

The results of the analysis performed on overall RT showed that diabetic status, and daily steps inversely predicted reaction speed (*R*^2^ = 0.16; diabetic status: std β = 0.29, *t* = 2.15, *p* = 0.038; daily steps: std β = -0.27, *t* = -2.09, *p* = 0.043), after accounting for the significant prediction accrued by BMI (*R*^2^ = 0.16, std β = 0.30, *t* = 2.21, *p* = 0.033). Diabetics showed longer RTs than their non-diabetic counterparts (**Figure 8A**), but a similar, inverse relationship between higher PA level and shorter RT (**Figure 8B**). The relationship between BMI and RT was direct, with a higher weight predicting longer RT in both diabetics and their non-diabetic counterparts, who marginally (*p* = 0.06) differed in BMI (diabetics: 29.4 ± 4.4; non-diabetics: 27.3 ± 4.43). *Post hoc* observed power (1-β) was 0.99. Moreover, to estimate if the absence of interaction between diabetic status and daily steps reflected truly independent effects, or a lack of power (Stone-Romero et al., 1994), a further *post hoc* power analysis for differences between slopes in moderated regression with diabetic/non-diabetic status as a dichotomous moderator was computed. Its low value (0.26) indicated lack of power.

#### Attentional Orienting Effects, Switch Costs, and Error Rates

Although descriptive statistics show noticeable differences in zooming out effects and local-to-global switch costs, regression analyses did not reveal any significant prediction of RT difference and accuracy variables accrued by health status and/or PA level. *Post hoc* observed power (1-β) from the analysis of zooming out and zooming in effects, local-to-global and global-to-local switch costs was 0.46, 0.49, 0.61, and 0.42, respectively.

## Discussion

The present study aimed to investigate the independent and interactive effects of aging and objectively measured PA levels on performance of the orienting and executive control networks in community-dwelling aging individuals and diabetics. In sum, the results show a pattern of effects suggesting that there is a generalized detrimental impact of aging on information processing speed, attentional effects, and performance accuracy. However, being physically active seems to partially dampen this age-related deterioration, exerting a protective effect on processing speed and on the ability to orient attention toward locations and objects in the visual field, as well as avoiding an age-related shift toward accurate, but slowed performance on the speed-accuracy trade-off. Instead, different from the general claim that PA is especially beneficial to executive function (Colcombe and Kramer, 2003; Gajewski and Falkenstein, 2016), physically active habits appear to neither outweigh, nor attenuate the detrimental effect of aging on the executive control processes involved in task set reconfiguration. Furthermore, diabetic status and PA level resulted to affect processing speed in opposite directions, whereas they did not affect the performance of the orienting and executive control networks as reflected in orienting effects and switch costs.

To our knowledge, this was the first study of PA effects on cognition in aging people to investigate the performance of the orienting and executive control networks in combination in one task. Previous research combining the investigation of different attentional networks have been performed only in the area of acute exercise research by adopting Posner and Petersen’s (1990) attention network test that combines in one task warning signals prior to targets (alerting), cues that direct attention toward potential target locations (orienting) and target stimuli surrounded by congruent or incongruent flankers (executive control; Huertas et al., 2011; Chang et al., 2015). Differently, the attentional test developed by Pesce et al. (2003) and used for the present study merges typical features of the spatial orienting paradigm (Chica et al., 2014) with hierarchically built visual objects that contain global or local target features (Navon, 1977). The use of direct and informative cues with different cue-target SOAs and a low percentage of misleading cues allows tapping the initially exogenous and then strategic control over attention according to the informative value of the cue and the attentional re-orienting following miscued targets, led by the dorsal and ventral networks, respectively (Corbetta and Shulman, 2002; Petersen and Posner, 2012). The initial blocked task instruction, followed by the random presentation of global and local targets allows tapping true executive processes of stable set maintenance and adjustment of executive control on a trial-by-trial basis to switch attention between global and local attending led by the cingulo-opercular and fronto-parietal networks, respectively.

First, older age predicted lengthened RT, but only in the case of low-active individuals (**Figure 4**). This is in line with evidence of generalized slowing of information processing speed at old age (Birren and Fisher, 1995) and results of previous aging studies performed with the present attentional paradigm, which showed faster reaction speed in older athletes than in sedentary co-aged individuals (Pesce et al., 2005, 2007a, 2011). However, results also suggest that from late middle-age to old adulthood there is a differential shift in speed-accuracy trade-off setpoint between low-active and high-active individuals. In fact, high-active late middle-aged individuals showed averagely longer RTs (**Figure 4**), but lower rates of responses to no-go trials (**Figure 7B**). The pattern of results was reversed at older age, since high-active individuals were faster in responding than their low-active counterparts, but made more response errors. It seems that with increasing age, low-active older adults trade speed for maintaining accuracy as a compensatory strategy (Spirduso et al., 2005), whereas high-active individuals trade accuracy for maintaining speed of performance.

High PA levels seem also to dampen the age-related decline of efficiency of the orienting system. It must be pointed out that the size of the RT differences computed to estimate orienting effects and switch costs has an opposite meaning. As regards the orienting (zooming) effect, it represents the difference in RT between validly and invalidly cued trials. A large RT difference means that the individual was able to strategically orient attention toward the cued area, thus shortening the RT to validly cued target and had to pay a RT cost in the rare cases of miscued targets. This ability seems relatively scarce in low-active individuals already at late middle-age, when high-active individuals instead show a preservation of orienting ability reflected in a higher orienting effect (**Figure 5A**). Nevertheless, the active lifestyle no longer seems to buffer the age-related deterioration in old adulthood. The negligible size of the zooming out effect is attributable to the fact older adults show a typical local attending deficit (Pesce et al., 2005) that lengthens RT particularly when local targets and the preceding cue are not presented foveally, as it is the case for RT on valid local cue-local target trials that was used as subtrahend for the computation of the zooming out effect (**Figure 2**). The huge interindividual variability in zooming out effect is therefore an indicator that some individuals succeeded in overcoming the typical age-related local attending deficit, thus showing a positive zooming out effect, but other did not. A similar, but non-significant trend emerged for the zooming in effect (**Figure 5B**).

The graphical representation suggests that those, who succeeded were high-active late middle-aged individuals. Since orienting effects are RT differences, the larger effect in high-active late middle-aged individuals would be meaningless if it would be paralleled by a corresponding increment in absolute RT. This was not the case, as they showed lowest RTs. This strengthens the interpretation that being physically active helps overcoming age-related attending deficits. Intriguing neuroimaging evidence, while confirming the hypothesis that gains in cardiovascular fitness lead to enhanced neural efficiency, also shows a unique relationship between coordination training at old age and increased activation in the visuo-spatial orienting network (Voelcker-Rehage et al., 2011). The counteracting effect of overall PA on the age-related decline of attention orienting performance found in the present study might be therefore attributable, at least in part, to the coordinative demands of being physically active, since our objective measure of overall PA tapped a variety of possible activities at workplace, sports, house holding.

However, in studies of aging effects on the attentional networks, the most pronounced deterioration has been reported for the executive control network (Mahoney et al., 2010), which is also reported to be the primary locus of the beneficial effects of PA and exercise (Etnier and Chang, 2009), particularly at old age (Colcombe and Kramer, 2003; Gajewski and Falkenstein, 2016). In the present study, we focused on cognitive flexibility, a core executive function needed to switch attention between tasks that we measured by means of local switch costs. In contrast to the orienting effect, whose size reflected the ability to exert top-down control over attention to adhere to task requirements, local switch costs represented the inability to adjust executive control flexibly according to the unpredictable need to switch between global and local attending. Thus, the higher the switch cost, the lower the efficiency of the executive control. This type of costs is indeed thought to reflect the executive processes needed to deactivate a previous task set in favor of the actually relevant one. Differently from many other aspects of executive function that are benefited by PA, this type of cost was not positively affected by the PA level of the participants, but only negatively by age (**Figure 6**). This age-related decline was observed only for local-to-global switch costs, because global-to-local switch costs were biased by interacting spatial orienting effects. The strong automatic capture of attention by small cues interfered with the allocation of attention to visual objects (Goldsmith and Yeari, 2003), overweighing the persistence of attention on the last attended object that should facilitate RT in the case of consecutive local target trials used as subtrahend for the computation of the global-to-local switch cost (**Figure 3**; Pesce and Audiffren, 2011).

In sum, our findings parallel and extend to overall PA the notion that regular participation in exercise training, regardless of exercise mode, facilitates reaction speed, but is uninfluential on local switch costs (Dai et al., 2013). In their study across the lifespan, Pesce and Audiffren (2011) found that age and sport expertise independently predicted lower switch costs, whereas we could not find any effect by PA level. Taken together, the result suggests that not PA *per se*, but the cognitive demands inherent in many sports may be the mediator of PA effects on the executive control networks and particularly the fronto-parietal network (Pesce, 2012). This hypothesis refers to the “cognitive component skills approach” (Voss et al., 2010), suggesting that sport-related cognitive expertise may transfer to sport-unspecific tasks requiring fundamental cognitive abilities. The interpretation of the absence of PA effects on switch costs in the present study is in accordance with the finding that not PA, but cognitive training interventions in aging seem to have the potential to positively impinge on the plasticity of those specific processes and underlying neural substrate responsible for task switching ability (Gajewski and Falkenstein, 2012).

The second aim of the study was to investigate whether in aging diabetics, who are at risk of poor cognition and especially executive dysfunction (Qiu et al., 2006; Okereke et al., 2008; Luchsinger, 2012), a physically active lifestyle counteracts the deterioration of the ability to exert executive control over attention, which is particularly relevant for this special population. The outcomes of this study do not show impairments of specific aspects of the attentional networks performance as compared to non-diabetic co-aged participants, but only a worse information processing speed (**Figure 8A**). A physically active lifestyle seems beneficial to their processing speed to the same extent as it benefits performance of non-diabetic aging individuals. This means that the vascular pathologies that characterize the diabetic status and may be responsible for cerebrovascular disease and cognitive dysfunction (Luchsinger, 2012; Umegaki, 2014) can be counteracted – at least in terms of efficiency of the processes responsible for perceiving and responding – by physically active habits. Instead, the question if an active lifestyle counteracts the deterioration of the attention networks performance in diabetics needs further exploration, since this study resulted underpowered for that type of variables.

The study has further limitations that must be addressed. Merging the spatial orienting paradigm with the task switching between global and local stimulus features has the advantage to tap different attention networks with one task, but also led to some biases. One reason for the absence of PA effects on switch costs might be the relatively small size of such costs probably due to the presence of a spatial cue to switch. This anticipated information, typical of cueing paradigms, generally results in smaller switch costs (Wasylyshyn et al., 2011). This reflects an influence of the orienting network on the executive network, with the latter taking advantage from the information provided by the first to revolve a conflict and switch sooner (Callejas et al., 2005). Furthermore, the spatial cueing with small cues narrowed and captured attention, overweighing the global-to-local switch effect (Goldsmith and Yeari, 2003; Pesce and Audiffren, 2011). It is therefore possible that in our study, due to the influence of the advance cues on local switch costs, benefits by PA level could be detected only for attention orienting.

Thus, an outlook for future research is to use an interactionist approach to the study of PA effects on the attentional networks in aging (Callejas et al., 2005). The present study assessed local switch costs in heterogeneous blocks of spatially cued trials, but did not consider global switch costs in homogeneous trial blocks. Instead, beneficial PA effects in aging could be found for both local and global switch costs in uncued task switching (Hillman et al., 2006; Themanson et al., 2006). An interactionist approach with/without spatial cueing in both heterogeneous and homogeneous blocks of trials might further our understanding of whether the orienting of attention, preserved by an active lifestyle at least in late middle-age, is able to raise the efficiency of the executive control networks responsible for the adaptability of top-down control on a trial-by-trial basis and the stability of top-down control for set maintenance, respectively (Dosenbach et al., 2008).

A reason that may have prevented to detect a buffering effect of PA on the attentional performance of diabetics, as instead found for the orienting performance of non-diabetics, is the relatively small sample size and the intrinsically low power of moderated multiple regression analysis, particularly when the moderator is a dichotomous variable (Stone-Romero and Anderson, 1994; Stone-Romero et al., 1994). Extending the sample of diabetic elderlies can help distinguish true absence of PA effects on the attentional networks from power and generalizability issues of this convenience sample.

## Conclusion

Adequate levels of PA may positively influence the brain processes and systems responsible for information processing speed and the strategic control of the orienting of attention, but seem uninfluential on the ability to exert executive control for switching attention, which, instead, seems positively influenced by participation in cognitively demanding sports (Pesce and Audiffren, 2011). The differential association of physically and/or cognitively challenging activities to different attentional functions may provide the basis to design interventions for successful attentional aging tailored to exploit the multifaceted nature of the concept of an enriched environment, including PA and challenging cognitive tasks (Hertzog et al., 2009; Kraft, 2012). Given the broad range of unstructured, daily-life activities and structured exercise or grassroots/competitive sports composing overall PA levels measured in this study, our results add to the evidence that both daily-life PA as walking (Yaffe et al., 2001; Abbott et al., 2004) and sports participation (Pesce et al., 2007a; Zhao et al., 2016) may act as protective factors against cognitive decline in elderlies. These two main components of an active lifestyle (Condello et al., 2016) should be promoted by actions that impact the built environment to render it more conducive to PA (Saelens and Handy, 2008) and support physically active habits and sport participation until old age (Baker et al., 2010).

## Author Contributions

GC: Data acquisition with relevant role in data acquisition coordination, analysis and interpretation and drafting of the work, final approval of the version to be published and agreement to be accountable for all aspects of the work. RF: Data interpretation, drafting and critical revision of the work for important intellectual content with specific contribution as regards aging issues, final approval of the version to be published and agreement to be accountable for all aspects of the work. SF: Data acquisition and analysis, contribution to drafting the work, final approval of the version to be published and agreement to be accountable for all aspects of the work. JS: Interpretation of data and critical revision of the work for important intellectual content, final approval of the version to be published and agreement to be accountable for all aspects of the work. ADB: Interpretation of data and critical revision of the work for important intellectual content, final approval of the version to be published and agreement to be accountable for all aspects of the work. LC: Contribution to conception of the work with relevant role in project coordination, critical revision of the work, final approval of the version to be published and agreement to be accountable for all aspects of the work. CP: Main role in the conception and design of the work, creation of the attentional test, data analysis and interpretation, drafting of the work with specific contribution as regards the physical activity-attention relationship, final approval of the version to be published and agreement to be accountable for all aspects of the work.

## Conflict of Interest Statement

The authors declare that the research was conducted in the absence of any commercial or financial relationships that could be construed as a potential conflict of interest.
